# Compulsory care of individuals with severe substance use disorders and alcohol- and drug-related mortality: A Swedish registry study

**DOI:** 10.3389/fpsyt.2023.1106509

**Published:** 2023-01-18

**Authors:** Simone Scarpa, Robert Grahn, Lena M. Lundgren

**Affiliations:** ^1^Department of Social Work, Umeå University, Umeå, Sweden; ^2^Graduate School of Social Work, University of Denver, Denver, CO, United States

**Keywords:** compulsory care, addiction treatment, alcohol-related mortality, drugs other than alcohol-related mortality, addiction severity index (ASI)

## Abstract

**Aim:**

This study used 17 year of Swedish registry data (2003–2019) for 25,125 adults assessed for their severity of substance use to identify the baseline factors predicting the risk of being court-ordered into compulsory care and examine the association between admission to compulsory care and mortality risks due to alcohol- or drug-related causes.

**Methods and materials:**

Addiction Severity Index (ASI) assessment data were linked to register data on demographic characteristics, compulsory care, and alcohol- and drug-related mortality. Cox regression models were used to identify baseline factors predictive of post-assessment admission to compulsory care in the 5 years post-substance use assessment. Discrete-time random-effect logistic regression models were used to examine the association between compulsory care duration and alcohol or drug-related mortality risks. Propensity score matching was used for validation.

**Results:**

The first models identified that younger age, female gender, and ASI composite scores for drug use, mental health and employment were significantly associated with the risk of placement in compulsory care for drugs other than alcohol. Female gender and ASI composite scores for alcohol, drug use and employment were significantly associated with compulsory care treatment for alcohol use. The second models showed that older individuals and men were more likely to die due to alcohol-related causes, while younger individuals and men were more likely to die due to drug-related causes. Length of stay in compulsory care institutions significantly increased the likelihood of dying due to substance use-related causes. Propensity scores analyses confirmed the results.

**Conclusion:**

In Sweden, a significant concern is the higher likelihood of women and young individuals to be court-ordered to compulsory care. Although compulsory care is often advocated as a life-saving intervention, our findings do not provide strong support for this claim. On the contrary, our findings show that admission to compulsory care is associated with a higher risk of substance use-related mortality. Factors such as compulsory care often not including any medical or psychological therapy, together with relapse and overdose after discharge, may be possible contributing factors to these findings.

## 1. Introduction

There is a strong association between having a severe substance use disorder and a range of problems in other life domains, such as physical or mental health issues, impairment in social relations, risk of engaging in criminal behavior, housing instability, employment, and financial problems (1). This substance use-related biopsychosocial vulnerability may in worst case lead to premature death. Many studies have in fact identified a relationship between severe substance use disorders and increased mortality risk (2–7). A way to mitigate this vulnerability, and therefore reduce the risk of premature death, is to offer substance use disorder treatment (5). In many countries, when individuals with substance use severe enough to put themselves or others in danger refuse to undergo voluntary treatment, they can be mandated to compulsory care, usually through a court order (8).

Sweden is one of the countries where courts can order compulsory care for individuals with severe alcohol and or drug use disorders. Compulsory care for severe substance use is relatively common in Sweden and about 1,000 people per year are court-ordered to the treatment (8–13). Individuals who are admitted to compulsory care for severe substance use tend to have more severe alcohol and drug use disorders as well as to be younger and more socially disadvantaged compared with those engaging in voluntary treatment options (14, 15). Moreover, individuals self-reporting a history of compulsory care treatment for severe substance use are more likely to have greater treatment needs, more substance use related-problems and experience more social exclusion (e.g., from social relationships, or related to unstable housing and employment, or due to various forms of discrimination), compared with those with only a history of voluntary treatment (14–17).

Previous studies have focused on the association between compulsory care for severe substance use and mortality (18, 19). These studies compared the mortality risks of patients who have been discharged from compulsory care for severe substance use and the general population or focused on within-group differences among those with a history of compulsory care. Their findings showed higher mortality risks for those who were required to undergo compulsory care for severe substance use, compared to the Swedish population as a whole. For example, the study by Hall et al. (18) found that between 2001 and 2009 compulsory care patients had a death rate between 8 and 10 times higher than the general population. Ledberg and Reitan (19) showed that those discharged from compulsory care for severe substance use, and young patients in particular, faced the greatest mortality risk within 2 weeks after the end of the treatment.

Yet, no previous study has investigated whether individuals with severe substance use disorders who have been mandated to compulsory care are more or less likely to die due to alcohol- or drug-related causes compared to those with similar substance use profiles who were not mandated to participate in treatment. To address this knowledge gap, this study: (1) Identified risk-factors associated with placement in compulsory care for risky substance use; and (2) examined whether placement in compulsory care was associated with either reduced or increased risk of substance use-related mortality after discharge. Addiction Severity Index (ASI) assessment data and register data on demographic characteristics, compulsory care admission and alcohol- and drug-related mortality were linked and analyzed to address these research questions.

## 2. Materials and methods

### 2.1. Study setting

In Sweden, individuals with substance use problems so severe to constitute a danger for themselves or others, and for whom voluntary treatment is deemed to be inadequate, can be legally mandated to compulsory care for substance use disorder. Sweden’s Care of Abusers (Special Provisions) Act (*Lag om vård av missbrukare i vissa fall*, or LVM) is founded on the framework of civil law, and not on a criminal justice framework (10). In comparison with the United States or other Nordic countries, where compulsory care for severe substance use typically takes place within the criminal justice system or within the psychiatric care system (10), Swedish compulsory care for substance use disorder is overseen and implemented by the Swedish National Board of Institutional Care (*Statens institutionsstyrelse*, or SiS). The National Board of Institutional Care is an independent Swedish government agency that has the legal right and responsibility to provide compulsory care in locked facilities to individuals who are deemed to require such treatment. It is possible to remain in compulsory care for up to 6 months, usually without participating in any medical or psychological therapy for substance use disorder. This means that it is virtually possible to stay in compulsory care without receiving any treatment for substance use problems. Patients are only required to remain abstinent from alcohol and drug use during this period. The decision to terminate the treatment is taken jointly by the compulsory care institution and the municipal social service board that required the treatment. Municipal social service boards are legally responsible for all decisions related to compulsory care admissions. This means that they are legally obliged to justify the need for treatment by determining that a person is at risk to oneself or others due to their substance use problems and is also unwilling to undergo voluntary treatment. Admission decisions are taken by the municipal social service boards but need to be submitted to regional administrative courts. Regional administrative courts take the final decision regarding compulsory care admissions. A policy concern in Sweden, but also in other countries with similar addiction treatment systems, is to assess whether mandatory treatment reduces mortality risks among individuals with risky and severe substance use.

### 2.2. Research questions

This study addressed two questions: (1) What baseline factors predicted the risk of being court-ordered into compulsory care within 5 years after being assessed for substance use severity? (2) Was admission to court-ordered compulsory care post-assessment of substance use severity associated with a reduced risk to die of alcohol or drug-related causes, compared to individuals assessed for substance use severity who were not admitted to compulsory care? Each of the questions was addressed using different survival analysis modeling approaches and different samples from the same data sources.

### 2.3. Data sources

This study uses ASI assessment data from 144 Swedish municipalities over the period 1999–2019. The individuals in the database represent approximately 40 percent of individuals who completed an ASI baseline interview in Sweden during the study period. The database is representative of the urban adult population with ASI-assessed problems, with an underrepresentation of smaller and rural municipalities. The use of the ASI as an assessment tool for substance use disorders and associated problems is common in Sweden. About 93 percent of the Swedish population live in municipalities where social workers use the ASI tool. Social workers’ training to use the ASI tool is supervised at the national level by the Swedish National Board of Health and Welfare (NBHW, or Socialstyrelsen), which provided us with the original data. Patients are often self-referred to ASI-assessments, but they can also be referred by primary and secondary health-care services, police and court officials, or through family members and other venues.

The baseline ASI survey was linked to three other databases using pseudonymized individual identifiers: (1) Data on adults court-mandated to compulsory care for their substance use problems, which come from the registers of the Swedish National Board of Institutional Care (SiS, or *Statens institutionsstyrelse*); (2) data from the Swedish Causes of Death Register, maintained by the NBHW; (3) data from Swedish population registers containing demographic information on age, gender, country of birth and date of emigration, maintained by Statistics Sweden.

The NBHW, the Regional Ethical Review Board at Umeå University (DNR: 2016/504-31; amendment 2020-06233) and Institutional Review Board (IRB) of the University of Denver reviewed and approved the study protocol. All study data were de-identified and the study met criteria for IRB exemption.

### 2.4. Study design and population

We use two different samples from the same data sources for our study. The first sample was created to address our first research question (i.e., what baseline factors predicted the risk to be court-ordered into compulsory care). From the ASI-database, we selected all adults (18 years of age and older) with complete demographic data (*N* = 14,395) who were assessed for substance abuse disorders between 1999 and 2014 and did not have a history of emigration or die in the 5 years following the assessment. We linked these data to register data for compulsory care entries in the 5 years post-assessment to identify the background factors associated with the likelihood of admission to treatment.

To address the second research question (i.e., whether entry into court-mandated compulsory care is associated with mortality due to alcohol or drug-related causes), we created a larger sample including all adult individuals who completed an ASI-assessment between 1999 and 2019 (*N* = 25,125), excluding those who emigrated during the study period. We linked these data to the nationwide register data for compulsory care and to the causes of death register to assess the association between admission to compulsory care and alcohol- and drug-related mortality.

### 2.5. Outcome variables

The dependent variable for the two models addressing the first research question was entry into court-mandated compulsory care. We distinguished two types of compulsory care, based on the type of substance use disorder being treated: Compulsory care for alcohol-use disorder and compulsory care for drug-use disorder. The two outcomes were categorized as a yes/no dichotomous variable and were not mutually exclusive. In fact, about one fifth of compulsory care patients in our sample (192 cases out of *N* = 931 cases) received treatments for both alcohol and drug problems and were thus included in both categories.

The two dependent variables for the models addressing the second research question were alcohol-related mortality and drugs other than alcohol, related mortality. Alcohol-related mortality and drugs other than alcohol related mortality were categorized as yes/no dichotomous variables, derived from the NBHW Causes of Death Register. Causes of deaths were determined by the NBHW based on ICD-10 codes. Alcohol-related death was defined as having either an underlying or contributing cause of death related to alcohol, such as, for example, alcoholism, toxic effect of alcohol or mental and behavioral disorders due to the use of alcohol. A death is considered drug-related if a drug played a role in the death, either directly or indirectly (20). The two outcomes were not mutually exclusive because 6.6 percent of the individuals with substance-related death died from both alcohol- and drug-related causes (92 cases out of *N* = 1,390 cases).

### 2.6. Covariates

Control variables were based on answers reported at the ASI baseline assessment or on the other register databases used in the study. The variable *Age* was recoded into a categorical variable with 7-year age bands up to age 24, then three 10-year age bands (25–34, 35–44, and 45–54), with a last age band for all those 55 and older. Prior studies based on Swedish data found that the profiles of substance use disorders differ by age group, with alcohol use disorders being more common among older individuals (21) and drug use problems more common among younger cohorts (22).

The *Gender* variable was a dichotomous variable, with male as the reference category, and female assuming a value of one. This variable refers to biological sex and one limitation of our study is that we are unable to identify individuals who identify themselves as non-binary or transgender.

*Immigrant background* was a five-category variable considering country of origin and distinguishing first- and second-generation immigrants: Individual and their parents all born in Sweden (the reference group); individual born in Norway, Finland, or Denmark (first-generation immigrant); individual born outside of Sweden, Norway, Finland, or Denmark (first-generation immigrant); individual born in Sweden with at least one parent born in Norway, Finland, or Denmark and no parent outside of Nordic countries (second generation immigrant); and individual born in Sweden with at least one parent born outside Nordic countries (second-generation immigrant). In Sweden, about every fifth person is a first- or second-generation immigrant. Unlike other countries, Swedish public authorities are not allowed to collect data on race or ethnicity, and they are only allowed to collect data on country of birth. Prior studies have identified significant differences by immigration status in alcohol or drugs other than alcohol related mortality, with individuals from non-Nordic countries being significantly less likely to die of such causes (7).

The ASI composite scores for severity of alcohol, drug, mental health, health, family and social relationships, employment, and legal problems were numeric variables with higher values indicating more problems/needs in the specific area (23, 24). Each ASI CS is an index computed from answers to questions related to an ASI problem area. As recommended by the developers of the ASI CSs (24), equal weighting is given to all questions/items within an ASI CS and each score is adjusted for the answer range of each item and the total number of items in the composite. The answer to each question is then divided by the highest possible response, and by the total number of questions in the ASI CS. The reliability of ASI CSs has been rigorously explored and tested by many studies carried out in different countries (25, 26). For example, recent studies based on Swedish ASI-data indicated that the ASI CS for mental health was a significant predictor of future inpatient hospitalization for mental health disorder (27) and the ASI CS for legal problems was a significant predictor for future imprisonment (28).

The main explanatory variable for the models addressing the second research question was days in compulsory care per year, recoded into hundreds of days. The variable is derived from the SiS register database and spells occurring over multiple calendar years were split to assign days to their corresponding calendar year.

### 2.7. Statistical analysis

As a first step, we examined descriptive statistics of the study sample. We stratified individuals by whether they were admitted to compulsory care at least once during the course of the study period, after ASI-assessment, and addressed differences in the control variables between those who were admitted to compulsory care and those who were not. Mean and percentages were analyzed for significance using student *t*-test or chi-square test, as appropriate.

In the second step, two Cox proportional hazards models were fit to identify the baseline factors differentiating those who were court-ordered into compulsory care from those who were not. The first model included admission into compulsory care for alcohol-use disorder as a dependent variable, while the dependent variable in the second model was admission into compulsory care for drug-use disorder. Based on a subset of individuals (*N* = 14,395) who were ASI-assessed between 1999 and 2014, we considered the first admission to either type of compulsory care within the 5 years post-ASI assessment (i.e., we did not consider entries after the first one or entries occurring after the time-range examined here). We excluded individuals who emigrated or died within the 5 years post-ASI-assessment. Since individuals could be assessed and court-ordered into compulsory care multiple times during the study period we adjusted standard errors to account for clustering of repeated observations within individuals using the *vce(cluster id)* option within the *stcox* command in Stata. We tested proportional hazards by testing for the independence between the scaled Schoenfeld residuals and the time-at-risk. The tests showed that only one variable violated the proportional hazard assumption, i.e., the ASI CS for drug in the model for entry into compulsory care for alcohol-use disorder. Therefore, this variable was included in the Cox model for entry intro alcohol-related compulsory care as a time-varying covariate interacted with time.

In the third step, our aim was to analyze the association between compulsory care and substance use-related mortality. We used all observations in the ASI-database (from 1999 to 2019) and individuals were followed until date of mortality or, for those surviving, through the end of the study period (i.e., 31 December 2019), at which point their event history was right-censored. Individuals who emigrated from Sweden during the study period were excluded from the analyses, resulting in *N* = 25,125 individuals. Our main independent variable, i.e., time in compulsory care, is time varying and indicates the cumulative number of days of treatment for that year since entry into the study (i.e., the first ASI-assessment). We applied discrete-time event history analysis with logistic estimation, because this a form of event history analysis that is appropriate for the investigation of the probability of the occurrence of our event of interest (substance-use related mortality), conditional on both time-varying and time- constant variables which may influence the probability of the specified event occurring (29). An advantage with discrete-time even history analysis is that we can treat compulsory care as an intermediate event, between ASI-assessment and survival or death, by defining it as a time-varying covariate. Two separate models were specified: the first model examines the association between the yearly cumulative duration of compulsory care spells and alcohol-related mortality while the second model examines the association between the yearly cumulative duration of compulsory care spells and drugs other than alcohol related mortality. In both models, duration of compulsory care is measured in days. We arranged our dataset in person-year format, with individuals contributing as many observations as the number of years they have been at risk of experiencing the risk in question, i.e., from the year of the first ASI assessment through to the year of last observation (or death). Therefore, after ASI assessment, individuals were prospectively followed-up for a mean (SD) of 7.2 (4.2) years and, for each year, it was reported the cumulative number of days in compulsory care (rescaled in hundreds). After the exclusion of missing data, our analytical sample contained 181,455 person-year observations for *N* = 25,125 individuals. For each individual, and in each year, we defined two dichotomous dependent variables, measured yearly, for the two types of substance-related death, with categories “death” and “survival” corresponding to the two possible outcomes each year. We defined the unit of analysis as a year because life duration is typically generally rounded to the last birthday, rather than reported as an exact age (e.g., in months or days). We believe that this time unit provide sufficient detail to analyze the effect of compulsory care on substance-use related mortality. A finer time unit (e.g., months) would not add substantively to our analyses and would make our sample computationally unfeasible. Year was recoded as an ordinal variable (with the assessment year = 1) and both models control for year of observation. Another advantage with discrete-time logistic models is that they can include a random effect term for individuals to account for individual-specific, time invariant unobservable characteristics (30). The presence of unobserved heterogeneity at the individual level is assessed by performing a likelihood ratio test for the intraclass correlation (rho). The test is significant in both models, implying that subject-level random effects explain part of the variance between individuals (i.e., we can reject the null hypothesis of rho = 0). We used a random effect specification instead of a fixed effect model because most of our variables are time-invariant, whose effect cannot be estimated by fixed effect models. Fixed effect models would only use data on individuals whose values on the outcome variables change over time (i.e., individuals who died due to alcohol- or drug-related causes during the study period), hence ignoring most of the data. Estimates based on this highly selected subset of individuals could not be generalized to the rest of the sample. Therefore, we use random-effect models also because these models do not imply a selection based on the outcome variables but make use of the entire sample.

As mentioned before (section 2.4. Study design and population), the outcomes for the models for both research questions are not mutually exclusive and this prevented us from adopting a competitive risks framework in our survival analyses.

As a sensitivity analysis, we created two propensity-score matched samples to balance baseline characteristics between individuals with and without post-assessment compulsory care during the study period. The variables for the propensity score matching were selected based on the results of the Cox regression models for entry into the two types of compulsory care. Different iterations of the propensity models were run using different model specifications and different nearest neighbor ratios by executing the Stata command *psmatch2* (31). We chose a 10-nearest neighbor matching because this matching algorithm ensured sufficiently large validation datasets for precise estimates, while still keeping bias (i.e., the difference in the mean of covariates between the groups with and without compulsory care) as low as possible. Next, as a robustness check, we created person-year datasets selecting the propensity score matched cases and re-run the discrete-time random-effect logistic models for these subsamples (10,310 individuals for the model for alcohol-related mortality and 10,032 individuals for the model for drug-related mortality). Our results were robust to the choice of different nearest neighbor matching algorithms (results available on request).

Stata version 17 was used for all calculations (StataCorp, College Station, TX, USA). Minimum statistical significance was set at *p*-value < 0.05 for all statistical analyses.

## 3. Results

Descriptive statistics are displayed in Table 1. We show only the descriptive statistics for the larger sample because percentages and means were essentially equivalent for the two samples. The proportion of individuals who died due to either cause was higher among those who entered compulsory care (13.5 percent) than among those who did not (5.4 percent), and the difference between the two groups was significant (*p* < 0.001). About 70 percent of the sample was men but the proportion of women was significantly higher (35.8 percent) in the compulsory care group (*p* < 0.001). Age also differed significantly between the two groups, measured either as categorical or continuous measure (*p* < 0.001). Those who were admitted to compulsory care were a younger population than their counterparts, with those in the age group 18–24 comprising 33.6 percent among those entering compulsory care after ASI-assessment, while they accounted for 18.9 percent of the total sample. Native-born individuals were slightly overrepresented and non-Nordic immigrants were underrepresented among those admitted to compulsory care. When looking at the ASI composite scores those admitted to compulsory care report more severe problems for all domains, except for alcohol. Finally, the average cumulative duration of treatments, until the end of the observation period, was 219.2 days for those admitted to compulsory care (albeit with a high SD: 175.8 days).

**TABLE 1:** Descriptive statistics in relation to admission to compulsory care.

Variable	With at least a compulsory care spell post-ASI assessment (*N* = 1,496), % or mean (±SD)	Without any compulsory care spell post-ASI assessment (*N* = 23,629), % or mean (± SD)	Total (*N* = 25,125), % or mean (± SD)
Status at the end of the study period***			
Still living	86.5	94.6	94.1
Deceased due to alcohol-related causes	5.3	2.7	2.9
Deceased due to drug-related causes	7.2	2.4	2.7
Deceased due to alcohol- and drug-related causes	1.0	0.3	0.4
Age at ASI assessment***	39.3 (13.8)	33.8 (12.8)	39.0 (13.8)
Age group***			
18–24	33.6	17.9	18.9
25–34	27.3	25.1	25.2
35–44	15.6	19.6	19.3
45–54	14.9	20.7	20.4
+55	8.6	16.7	16.2
Gender***			
Male	64.2	70.7	70.3
Female	35.8	29.3	29.7
***Immigrant background***			
Individual and their parents born in Sweden	72.5	69.5	69.7
Individual born in either Norway, Denmark, or Finland	3.4	3.7	3.7
Individual born outside of Sweden, Norway, Denmark, and Finland	6.7	10.7	10.5
Individual born in Sweden and at least one parent born in Norway, Denmark or Finland (no other country outside Sweden)	9.8	8.0	8.1
Individual born in Sweden and at least one parent born outside Sweden, Norway, Denmark, and Finland	7.6	8.0	8.0
***ASI composite scores at the assessment***			
Health	0.38 (0.34)	0.36 (0.34)	0.36 (0.34)
Employment***	0.87 (0.23)	0.77 (0.29)	0.77 (0.29)
Alcohol**	0.29 (0.29)	0.32 (0.29)	0.32 (0.30)
Drug***	0.16 (0.15)	0.11 (0.13)	0.11 (0.13)
Legal***	0.17 (0.23)	0.13 (0.21)	0.13 (0.21)
Mental health*	0.36 (0.24)	0.34 (0.24)	0.34 (0.24)
Family and social relations**	0.29 (0.23)	0.27 (0.22)	0.27 (0.22)
Days in compulsory care until the end of the study period***	219.2 (175.8)	0.0 (0.0)	13.1 (67.3)

*p < 0.05, **p < 0.01, and *** *p* < 0.001.

Table 2 shows the results of Cox regression models for the likelihood of entering compulsory care for either alcohol-use or drug-use disorders for individuals assessed for addiction severity between 1999 and 2014, within 5-years after their ASI-assessment. Results are presented as hazard ratios with 95 percent confidence intervals, indicating a change in the outcome variable (i.e., in the risk of entering compulsory care) given a change in the covariate. Depending on the type of the covariate (i.e., categorical or continuous), hazard ratios either compare the risk of one group to another group or compare the risk after a change in the continuous covariate to the risk at its original value. The *Age group* variable is significant only in the model for compulsory care for drug-use disorder and indicates that the risk decreases with age. In fact, all age groups have a lower risk to end up in compulsory care for drug-use disorder than do those aged 18–24, and the risk is extremely low for those over 55 years of age (*HR* = 0.10; 95% *CI* = 0.04–0.21). Hence, in Sweden drug-related compulsory care seems to be a coercive measure targeted especially at young people, whereas age is not significant when looking at the risk of entering compulsory care for alcohol use disorder. Regarding the *Gender* variable, women were more likely to be court-ordered to compulsory care than men, regardless of the substance use disorder to be treated. Compared to men, women had 37 percent increase on the risk of entering alcohol-related compulsory care (*HR* = 1.37; 95% *CI* = 1.11–1.71) and 39 percent increase on the risk of ending up in drug-related compulsory care (*HR* = 1.39; 95% *CI* = 1.15–1.69). Looking at the *Immigrant background*, first-generation immigrants from countries other than Northern Europe had a 44 percent lower risk to enter compulsory care for alcohol-related problems (*HR* = 0.56; 95% *CI* = 0.35–0.87) and a 37 percent lower risk of compulsory care for drug-related problems (*HR* = 0.63; 95% *CI* = 0.45–0.87), compared to the Swedish-born reference group. Immigrants from other Northern European countries had a 70 percent lower risk to enter drug-related compulsory care compared to Swedish-born (*HR* = 0.30; 95% *CI* = 0.09–0.94). The *ASI CSs* for employment, alcohol- and drug-use were significant in the model for entry into compulsory care for alcohol use disorder. Unsurprisingly, the ASI CS for alcohol was the strongest positive predictor in the model (*HR* = 3.93; 95% *CI* = 2.85–5.44), followed by the ASI CS for employment (*HR* = 2.86; 95% *CI* = 1.87–4.39). The hazard-ratio for the time-dependent variable for the ASI CS for drug is statistically significant and higher than one, indicating that the hazard for entry into alcohol-related compulsory care tends to increase over time, from the ASI-assessment, for those with a high score for drug use. This can be interpreted as a higher risk of alcohol-related compulsory care for individuals with an alcohol use disorder as main diagnosis upon assessment but who also are polysubstance users. In the model for drug-related compulsory care, the hazard ratios for the ASI CSs for drug use, employment, mental health, and alcohol are significant. As expected, the strongest positive predictor for the model was the ASI CS for drug use disorder (*HR* = 48.20; 95% *CI* = 24.13–96.28). Individuals with high scores for alcohol use disorder are less likely to be court-ordered into this type of compulsory care because they are more likely to end up in the other type of compulsory treatment for alcohol-related problems. Patients in compulsory care for drug use disorders are also more likely to have mental health problems (*HR* = 1.56; 95% *CI* = 1.02–2.40).

**TABLE 2:** Multivariate Cox regression models for entry into compulsory care for alcohol-use or drug-use disorders.

Independent variables	Model 1: Compulsory care for alcohol-use disorder	Model 2: Compulsory care for drug-use disorder
***Age group***		
18–24	1 (Reference)	1 (Reference)
25–34	0.86 (0.63–1.19)	0.63 (0.51–0.77)***
35–44	0.81 (0.59–1.12)	0.31 (0.23–0.41)***
45–54	0.90 (0.67–1.23)	0.23 (0.16–0.33)***
>55	0.80 (0.55–1.17)	0.10 (0.04–0.21)***
***Gender***		
Male	1 (Reference)	1 (Reference)
Female	1.37 (1.11–1.71)**	1.39 (1.15–1.69)***
***Immigrant background***		
Individual and their parents born in Sweden	1 (Reference)	1 (Reference)
Individual born in either Norway, Denmark or Finland	1.03 (0.66–1.60)	0.30 (0.10–0.94)*
Individual born outside of Sweden, Norway, Denmark, and Finland	0.56 (0.35–0.87)*	0.63 (0.45–0.87)**
Individual born in Sweden and at least one parent born in Norway, Denmark or Finland (no other country outside Sweden)	1.34 (0.99–1.81)	1.14 (0.86–1.51)
Individual born in Sweden and at least one parent born outside Sweden, Norway, Denmark, and Finland	0.78 (0.50–1.22)	0.88 (0.65–1.18)
***ASI-composite scores***		
Mental health	0.67 (0.42–1.07)	1.56 (1.02–2.40)*
Family and social relations	0.93 (0.56–1.53)	0.95 (0.59–1.51)
Employment	2.86 (1.87–4.39)***	5.45 (3.27–9.08)***
Alcohol	3.93 (2.85–5.44)***	0.52 (0.36–0.76)***
Drug use	0.00 (0.00–0.00)***	48.20 (24.13–96.28)***
Drug use X time (days)	1.00 (1.00–1.01)***	–
Health	1.26 (0.93–1.70)	1.27 (0.97–1.67)
Legal	0.65 (0.38–1.12)	0.82 (0.57–1.18)
Cases	14,395	14,395
Failures	413	518
Log-likelihood	−3476.23	−4118.65
pseudo-R2	0.02	0.07

Hazard ratios (with 95% confidence intervals). *p < 0.05, **p < 0.01, and ***p < 0.001.

The results of the discrete-time event-history random effect logistic models are shown in Table 3. The first model shows the association between days in compulsory care and alcohol related mortality, while the second model shows the association between days in compulsory care and drugs other than alcohol-related death. Both models control for demographic characteristics. The findings show that admission to compulsory care is significantly associated with higher odds ratios of substance use-related mortality. In fact, 100 days in compulsory care increased the odds ratio of dying due to alcohol by 48 percent (*OR* = 1.48; 95% *CI* = 1.34, 1.62) and the odds of dying due to drugs other than alcohol by 41 percent (*OR* = 1.41; 95% *CI* = 1.29–1.53). Based on these results, we can exclude that admission to compulsory care decreases the substance-related mortality risk of individuals who received an assessment for substance use severity.

**TABLE 3:** Discrete-time random-effect (RE) logistic models for dying of substance use disorder-related causes, 1999–2019.

Independent variables	Model 1: Alcohol-related mortality	Model 2: Drugs-related mortality
***Age group***		
18–24	1 (Reference)	1 (Reference)
25–34	1.34 (0.51–3.52)	0.96 (0.74–1.26)
35–44	4.86 (1.94–12.15)***	1.03 (0.78–1.36)
45–54	16.12 (6.58–39.48)***	0.64 (0.48–0.86)**
>55	32.99 (13.41–81.16)***	0.34 (0.25–0.47)***
***Gender***		
Male	1 (Reference)	1 (Reference)
Female	0.72 (0.60–0.86)***	0.43 (0.35–0.52)***
***Immigrant background***		
Individual and their parents born in Sweden	1 (Reference)	1 (Reference)
Individual born in either Norway. Denmark or Finland	0.92 (0.68–1.25)	0.96 (0.62–1.50)
Individual born outside of Sweden. Norway. Denmark and Finland	0.61 (0.43–0.87)**	0.54 (0.40–0.72)***
Individual born in Sweden and at least one parent born in Norway, Denmark or Finland (no other country outside Sweden)	0.87 (0.65–1.18)	1.06 (0.82–1.37)
Individual born in Sweden and at least one parent born outside Sweden, Norway, Denmark, and Finland	0.51 (0.39–0.83)**	0.87 (0.65–1.14)
*Days in compulsory care (in hundreds)*	1.48 (1.34–1.62)***	1.48 (1.29–1.53)***
Constant	0.00 (0.00–0.00)***	0.00 (0.00–0.01)***
Observations	181,455	181,455
Individuals	25,125	25,125
Log-likelihood	−4776.31	−4814.75
Wald Chi^2^	349.76***	201.19***
BIC	9697.93	21277.96
Random parameter σ_u_	1.29 (0.92–1.81)	1.11 (0.68–1.83)
Rho	0.33 (0.20–0.50)	0.27 (0.12–0.50)
LR test of rho	7.22**	3.05*

Reference category: Still alive. Odds ratios (with 95% confidence intervals). *p < 0.05, **p < 0.01, and ***p < 0.001. Both models control for year of observation.

With respect to the other covariates, we find that the likelihood of dying due to alcohol-related causes increased with age, keeping other things constant, and this variable is the strongest predictor of alcohol-related mortality. On the other hand, individuals in the two oldest age groups were less likely to die due to drugs other than alcohol than those in the youngest age group (44–55 years old: *OR* = 0.64; 95% *CI* = 0.48–0.86; >55 years old: *OR* = 0.34; 95% *CI* = 0.25–0.47). Taken together this suggests that the age profiles of those dying of drug-related causes are congruent with the age profiles of those who are also more likely to be admitted into compulsory care for drug addiction. The *Gender* variable was also a significant predictive factor for both types of mortality. Women were 28 percent less likely to die of alcohol-related cause (*OR* = 0.72; 95% *CI* = 0.60–0.86) and 57 percent less likely to die of drugs other than alcohol-related causes (*OR* = 0.43; 95% *CI* = 0.35–0.52) than men were. Hence, women were more likely to end up in both types of compulsory care but at the same time they were less likely to die either of due to alcohol- or drug-related reasons. This finding is in line with prior evidence from studies on gender differences in substance-related mortality conducted in Sweden and in other countries (32–34). With respect to *Immigrant background*, first-generation immigrants from outside Northern Europe (*OR* = 0.61; *CI* = 0.43–0.87) and second-generation immigrants with at least one parent born outside Northern Europe (*OR* = 0.51; *CI* = 0.31–0.83) had a lower risk of dying from alcohol-related causes, compared with the native-born. Individuals born outside the Nordic countries had also a lower likelihood of dying of drugs other than alcohol-related causes compared with individuals born in Sweden to parents born in Sweden (OR: 0.54; CI: 0.40–0.72). These findings confirm earlier research on the association between immigrant-background and substance use-related mortality in Sweden (7).

In order to account for potential selection bias due to the non-randomized nature of our data, we performed two propensity score matching analyses. The analyses were done to create treatment and control groups that were more similar in their baseline characteristics, allowing a more accurate assessment of the association between compulsory care duration and substance use-related mortality. Matching was done using logit propensity score nearest neighbor matching procedures. Selection of covariates was informed by the results obtained from the Cox models presented in Table 2. Both samples were thus matched on *Age*, *Gender*, and *Immigration Status*. The *ASI CS for alcohol* was entered in the propensity-score model for alcohol-related compulsory care, while the *ASI CS for drug* was entered in the propensity score model for drug-related compulsory care. Tables 4, 5 present the balance of the matching covariates according to treatment status (i.e., whether they received compulsory care or not) for two propensity-score-matched samples. After propensity matching, differences in covariates were substantially reduced between groups, compared to the original sample. Next, discrete-time event-history random effect logistic models were run on the matched samples to corroborate our findings. Table 6 shows that propensity score adjusted results were similar to the unadjusted analysis. The associations between compulsory care duration and alcohol-related mortality (*OR* = 1.53; 95% *CI* = 1.36, 1.72) and between compulsory care duration and drug-related mortality (*OR* = 1.41; 95% *CI* = 1.28, 1.55) were confirmed by the propensity score adjusted estimates.

**TABLE 4:** Balance of the matching variables in relation to alcohol-related compulsory care.

	Propensity-score-matched sample n. 1, % or mean (± SD)	
Matching covariates	With at least a compulsory care spell post-ASI assessment (*N* = 1,402)	Without any compulsory care spell post-ASI assessment (*N* = 8,908)
Age at ASI assessment***	33.8 (12.9)	36.0 (13.2)
***Gender***		
Male	64.2	66.1
Female	35.8	33.9
Immigrant background*		
Individual and their parents born in Sweden	72.8	69.4
Individual born in either Norway, Denmark, or Finland	3.4	3.5
Individual born outside of Sweden, Norway, Denmark, and Finland	6.3	8.7
Individual born in Sweden and at least one parent born in Norway, Denmark or Finland (no other country outside Sweden)	10.1	10.1
Individual born in Sweden and at least one parent born outside Sweden, Norway, Denmark, and Finland	7.6	8.3
ASI composite score for alcohol**	0.29 (0.29)	0.32 (0.29)

*p < 0.05, **p < 0.01, and ***p < 0.001.

**TABLE 5:** Balance of the matching covariates in relation to drug-related compulsory care.

	Propensity-score-matched sample n. 2, % or mean (± SD)	
Matching covariates	With at least a compulsory care spell post-ASI assessment (*N* = 1,451)	Without any compulsory care spell post-ASI assessment (*N* = 8,581)
*Age at ASI assessment*	33.8 (12.8)	34.5 (12.6)
Gender**		
Male	64.4	64.5
Female	35.6	35.5
Immigrant background***		
Individual and their parents born in Sweden	72.6	67.4
Individual born in either Norway, Denmark, or Finland	3.3	4.9
Individual born outside of Sweden, Norway, Denmark, and Finland	6.4	8.7
Individual born in Sweden and at least one parent born in Norway, Denmark or Finland (no other country outside Sweden)	10.1	11.0
Individual born in Sweden and at least one parent born outside Sweden, Norway, Denmark, and Finland	7.6	7.9
*ASI composite score for drug*	0.16 (0.15)	0.16 (0.14)

**p < 0.01 and ***p < 0.001.

**TABLE 6:** Discrete-time random-effect (RE) logistic models for dying of substance use disorder-related causes, 1999–2019 (with propensity score adjustment).

Independent variables	Model 1: Alcohol-related mortality	Model 2: Drugs-related mortality
***Age group***		
18–24	1 (Reference)	1 (Reference)
25–34	1.42 (0.40–5.04)	0.95 (0.68–1.33)
35–44	6.25 (1.85–21.12)**	1.13 (0.78–1.65)
45–54	20.11 (6.06–66.77)***	0.63 (0.42–0.97)*
>55	40.82 (12.06–138.16)***	0.42 (0.25–0.68)***
***Gender***		
Male	1 (Reference)	1 (Reference)
Female	0.70 (0.510.97)*	0.37 (0.28–0.49)***
***Immigrant background***		
Individual and their parents born in Sweden	1 (Reference)	1 (Reference)
Individual born in either Norway. Denmark or Finland	0.90 (0.52–1.55)	0.68 (0.35–1.33)
Individual born outside of Sweden. Norway. Denmark and Finland	0.41 (0.19–0.87)*	0.49 (0.30–0.79)**
Individual born in Sweden and at least one parent born in Norway, Denmark or Finland (no other country outside Sweden)	0.68 (0.40–1.16)	0.93 (0.66–1.32)
Individual born in Sweden and at least one parent born outside Sweden, Norway, Denmark, and Finland	0.32 (0.12–0.87)*	0.75 (0.49–1.16)
*Days in compulsory care (in hundreds)*	1.53 (1.36–1.72)***	1.41 (1.28–1.55)***
Constant	0.00 (0.00–0.01)***	0.00 (0.00–0.01)***
Observations	75,88	72,456
Individuals	10,310	10,032
Log-likelihood	−1719.83	−2365.68
Wald Chi^2^	142.30***	104.95***
BIC	3574.50	4865.66
Random parameter σ_u_	1.75 (1.27–2.44)	1.38 (0.90–2.12)
Rho	0.48 (0.33–0.64)	0.37 (0.20–0.58)
LR test of rho	9.47**	4.50*

Reference category: Still alive. Odds ratios (with 95% confidence intervals). With propensity score adjustment for alcohol-related compulsory care (Model 1) and drug-related compulsory care (Model 2). *p < 0.05, **p < 0.01, and ***p < 0.001. Both models control for year of observation.

## 4. Discussion

Our study identified two key findings. First, among adults assessed for substance use severity, there were significant differences between those who were court-ordered into compulsory care and those who were not required to participate in compulsory care. Those who were court-ordered to compulsory care for use of drugs other than alcohol were likely to be younger, have higher ASI CS for drug use and score higher on ASI CS for mental health and employment, while scoring lower on ASI CS for alcohol. Those who were court-ordered to compulsory care for alcohol were instead more likely to have higher ASI CS for alcohol compared to their counterparts, together with a high score for ASI CS for employment. ASI CS for drug also had a positive small effect for each year following assessment. For both types of compulsory care, women were more likely than men to end up in compulsory care. The second key finding of this study is that court-ordered admissions to compulsory care were significantly associated with higher odds of substance use-related mortality. This is a concerning finding for both scholars and practitioners since, in Sweden as in other countries with similar addiction-treatment systems, compulsory care for severe substance use is designed to reduce the risk of endangering oneself, after discharge, due substance use problems.

Other important findings are related to the higher likelihood of young adults and women among individuals placed in compulsory care for substance use severity. We can suggest some hypotheses to explain the overrepresentation of these groups. With respect to young adults, this finding may be due to a lower willingness to seek voluntary treatment, compared to older age groups. A first possible explanation for the overrepresentation of women could be that mothers tend not to seek voluntary treatment for addiction at an early stage of their substance use disorder, possibly due to concerns related to the risk of losing their children to the child welfare system. A second tentative explanation is that women are more likely to end up in compulsory care because their substance use problems tend to be evaluated as more severe than those of men by social workers. A third possibility is that women are given priority to this treatment, compared to men, due to the limited availability of other types of long-term care in Sweden. However, these hypotheses require further investigation in future studies.

## 5. Conclusion

Swedish compulsory care for severe substance use does not seem to be associated with a lower risk of substance use related mortality among individuals with risky substance use or substance use disorders. In fact, our study results point to the opposite. One possible reason for this is that, for many individuals placed in compulsory care for substance abuse treatment, the time in treatment corresponds to an imprisonment period in a locked care facility without any medical or psychological therapy. Accordingly, our findings support the recommendation that when compulsory care is deemed necessary, this type of care should include the highest quality of evidence-based care and supportive services to prevent a worsening of substance use problems after discharge. A second recommendation is the importance of providing access to both addiction treatment and psychiatric treatment during compulsory care, particularly for individuals placed in care due to drug other than alcohol use disorders. Previous studies have in fact found that this is a particularly vulnerable group and that worse mental health conditions at assessment were significantly associated with higher rates of mortality from suicide or where drug overdose was the primary or secondary cause of death (35).

### 5. 1. Limitations

One limitation of this study is that substance use severity was only measured by the ASI CSs for alcohol and drug use and we are unaware of the representativeness of our sample of individuals with ASI-assessed problems to all individuals with substance use problems in Sweden. Another limitation is that we do not have information on whether (and how) ASI CSs are used in decision making regarding being mandated to compulsory care and, by and large, about the possible long-term interactions between the assessment and the compulsory care systems. However, the ASI-interview is the most used assessment tool by Swedish social workers in order to assess substance use (and related) problems and plan interventions responding appropriately to the nature of these problems. Moreover, ASI composite scores have been rigorously tested for reliability and validity in many settings over the years and the results presented here corroborate previous findings about factors associated with higher odds of substance use-related mortality in Sweden (7, 25, 36, 28).

## Data availability statement

The register data used in this study cannot be made publicly available under Swedish privacy laws. Data for this research project are from the National Board of Health and Welfare and Statistics Sweden, which do not permit data-sharing according to the Swedish Secrecy Act. Investigators may apply to access the study data by contacting the Swedish National Board of Health and Welfare (registerservice@socialstyrelsen.se) and Statistics Sweden (mikrodata.individ@scb.se).

## Ethics statement

The NBHW, the Regional Ethical Review Board at Umeå University (DNR: 2016/504-31; amendment 2020-06233) and Institutional Review Board (IRB) of the University of Denver reviewed and approved the study protocol. Written informed consent from the patients was not required to participate in this study in accordance with the national legislation and the institutional requirements.

## Author contributions

SS: conceptualization, data curation, formal analysis, methodology, and writing—original draft preparation. RG: conceptualization and writing—original draft preparation. LL: conceptualization, funding acquisition, project administration, supervision, validation, and writing—review and editing. All authors contributed to the article and approved the submitted version.
